# The role of anxiety and gender in anticipation and avoidance of naturalistic anxiety‐provoking experiences during adolescence: An ecological momentary assessment study

**DOI:** 10.1002/jcv2.12084

**Published:** 2022-06-22

**Authors:** Ashley R. Smith, Emily L. Jones, Anni R. Subar, Quyen B. Do, Katharina Kircanski, Ellen Leibenluft, Melissa A. Brotman, Daniel S. Pine, Jennifer S. Silk

**Affiliations:** ^1^ National Institute of Mental Health, Emotion and Development Branch Bethesda Maryland USA; ^2^ Department of Psychology University of Denver Denver Colorado USA; ^3^ Department of Psychology University of Pittsburgh Pittsburgh Pennsylvania USA

**Keywords:** anticipation, avoidance, ecological momentary assessment, gender differences, pediatric anxiety

## Abstract

**Objective:**

Anxiety symptoms often increase in late childhood/early adolescence, particularly among girls. However, few studies examine anxiety‐relevant gender differences during anticipation and avoidance of naturalistic experiences during adolescence. The current study uses ecological momentary assessment (EMA) to examine associations among clinical anxiety, gender, anticipation, and attempted avoidance of person‐specific anxiety‐provoking experiences in youth ages 8–18.

**Method:**

124 youth (73 girls) completed 7 consecutive days of EMA. Seventy participants (42 girls) met criteria for one or more anxiety disorders, while the remaining 54 were healthy controls (31 girls). Participants reported the experience that they were “most worried about happening that day” and completed ratings about that event including whether they attempted to avoid that experience. Multilevel models examined whether diagnostic group (anxious, healthy), gender (boys, girls), or their interaction predicted anticipatory ratings or avoidance of these experiences.

**Results:**

Analyses revealed significant diagnostic group by gender interactions for anticipatory ratings. Specifically, anxious girls reported greater worry and predicted more negative outcomes related to future experiences. However, only a main effect of diagnostic group emerged for attempted avoidance. Finally, anticipatory worry predicted higher rates of attempted avoidance, but this association did not vary by diagnostic group, gender, or their interaction.

**Conclusion:**

These findings extend the literature on the interplay of anticipation and avoidance to person‐specific naturalistic experiences in pediatric anxiety. They reveal that anxious girls report more anticipatory anxiety and worry, while avoidance of real‐world anxiety‐provoking scenarios is a key concern for anxious youth independent of gender. By using EMA to examine person‐specific anxiety‐inducing experiences we can begin to understand how these processes and experiences unfold in the real world.

**Key points**

- Anxiety symptoms and diagnoses often increase in late childhood/early adolescence, particularly among girls. However, gender differences in key features of pediatric anxiety (i.e., negative expectancy, avoidance) do not consistently emerge.
- Using ecological momentary assessment (EMA) in youth ages 8–18, we found that anxious girls reported the highest levels of worry and expected future experiences to be most negative in anticipation of a person‐specific anxiety‐provoking experience. No gender differences emerged in rates of attempted avoidance, providing an exciting avenue for future research exploring potential predictors of avoidance that may vary by gender.
- These findings underscore the importance of understanding how anxiety‐relevant processes unfold in the real world, how these experiences may vary by gender, and the importance of assessing person‐specific experiences using temporally sensitive methods, such as EMA.

## INTRODUCTION

Anxiety disorders are common and debilitating disorders characterized by excessive worry and fear that often increases in late childhood/early adolescence, particularly among girls (Essau et al., 2018; Hartung & Lefler, 2019). Compared to non‐anxious individuals, individuals with anxiety expect future experiences to be more negative (e.g., McManus et al., 2000; Spielberg et al., 2015). Heightened anticipatory processes are linked to avoidance of potentially negative experiences (Grupe & Nitschke, 2013), a core feature of anxiety disorders. Given increases in anxiety symptoms during adolescence, particularly among girls, it is critical to identify patterns of behavior and emotional experiences that may help differentiate those who present with clinical levels of anxiety from typically developing youth.

Clinical data show stark gender^1^ differences in anxiety diagnoses, with females being twice as likely to meet criteria for an anxiety disorder as males (McLean et al., 2011), but gender differences in specific features of anxiety remain inconsistent. For instance, some studies report higher anxiety prior to potentially negative experiences in females (Behnke & Sawyer, 2000; McLean & Hope, 2010; Straube et al., 2009) while others fail to find any gender differences (Stoyanova & Hope, 2012). Similarly, gender differences in interpretation bias following negative, or ambiguous, experiences are also inconsistent (e.g., Gluck et al., 2014; Mobach et al., 2019; Stuijfzand et al., 2018). On the other hand, gender differences in avoidance (e.g., Klein et al., 2011; Lau et al., 2012) are more consistent. Thus, consistent gender differences exist in overall anxiety diagnoses, but inconsistent findings exist for particular anxiety features. Much research has focused on identifying potential mechanisms underlying gender differences in anxiety diagnoses, outlining many potential biological, psychological, and environmental factors that may account for stark differences in anxiety diagnoses (Altemus et al., 2014; Bangasser & Cuarenta, 2021; Craske, 2003). However, a widely accepted and supported model has yet to emerge.

Research is needed to compare particular features of anxiety across genders, especially during adolescence, when symptoms of anxiety increase. Further, retrospective rating scales possess limitations as measures of particular anxiety features. This is because affective scales are vulnerable to various forms of bias related to problems in recall over periods of a week or longer (see Robinson & Clore, 2002; Schwarz, 2012). In fact, in the anxiety literature, there has been much discussion and debate about whether gender differences in anxiety diagnoses and/or anxiety‐related distress reflect differences in retrospective reporting of emotions in males and females, with females more emotionally expressive regarding past events and thus more likely to report anxiety symptoms (for review see, Craske, 2003). In particular, there is evidence to suggest that males underreport anxiety symptoms (Egloff & Schmukle, 2004; Pierce & Kirkpatrick, 1992, although see; McLean & Hope, 2010). To reduce such biases, the current study utilized a smartphone‐based ecological momentary assessment (EMA) method to assess various anxiety features as they unfold in the real world. Such data could address debates concerning the degree to which gender differences relate to patterns of symptom‐reporting or behavior. We examine associations among clinical anxiety, gender, and core features of anxiety surrounding person‐specific anxiety‐provoking experiences in youth ages 8–18.

Theories suggest that worry about potential future negative outcomes (i.e., negative expectancy bias) leads anxious individuals to attempt to avoid such events or experiences (Grupe & Nitschke, 2013). The current study focuses specifically on “anticipatory anxiety” or “worry,” which concerns feelings evoked by uncertain threats that are not immediately present but may soon be encountered. This contrasts with the feeling of “fear,” generated by an immediately present threat (Davis, 1998). There is work linking anxiety, or worry, to avoidance in both children and adults (Bahrami & Yousefi, 2011; Dickson et al., 2012; Field et al., 2008; Field & Lawson, 2003). Avoidance is often considered a critical factor in the maintenance of anxiety disorders as it prevents new learning from occurring thus maintaining, or potentially increasing, future worry surrounding that event (for review see Krypotos et al., 2015). The most widely utilized treatment for pediatric anxiety, Cognitive Behavioral Therapy (CBT), focuses on decreasing avoidance and encouraging participants to experience their individualized anxiety‐provoking experiences to decrease future anxiety surrounding those experiences. Expanding our understanding of precursors to, or mechanisms of, avoidant behaviors in adolescence may therefore inform new targets/strategies for decreasing avoidance in anxious adolescents. However, most work in this domain is limited in scope (e.g., De Jong & Muris, 2002; Guyer et al., 2008; Muris et al., 2007; Smith et al., 2018) relying on retrospective reports, which are subject to recall bias, or standardized laboratory tasks, which possess other limitations. For instance, most tasks are not tailored to participants' daily concerns and utilize standardized procedures rather than experiences specific to each participant's concerns. In contrast, the current study examines the relation between reports of worry and avoidance surrounding anticipated real‐life experiences that participants are most worried about occurring that day.

Importantly, this type of real‐world study may also help diminish hypothesized, or documented (e.g., Pierce & Kirkpatrick, 1992, for review see; Craske, 2003) gender biases in the assessment of anxiety‐related phenomena by (1) reducing the time between the anxiety related experience and recall of that experience, and (2) focusing on individualized anxiety‐provoking experiences decreasing the likelihood that gender differences emerge due to the type of worry being assessed. Together, this approach increases the generalizability (i.e., experiences can vary amongst participants) and ecological validity (i.e., probing real world experiences) of any potential gender‐related findings.

### Current study

The current study uses smartphone‐based EMA to examine associations among clinical anxiety, gender, anticipatory ratings, and attempted avoidance of person‐specific anxiety‐provoking experiences in youth, ages 8–18, with and without a current anxiety disorder. EMA allows for examination of real‐world anxiety‐provoking experiences while reducing issues inherent to retrospective reporting. By prompting participants to answer questions about negative expectancy bias, magnitude of worry, and avoidance at multiple timepoints within a day, we can also assess temporal associations between anticipatory processes and avoidance, better informing mechanistic models. This assessment probes person‐specific anxiety‐provoking experiences, rather than general experiences that may or may not be shared across, or within, a specific disorder. Here, youth report circumstances evoking the highest level of worry for themselves that day (e.g., picking a table in the cafeteria, trying out for a sports team, talking to a peer) and answer questions related to that experience throughout the day. Importantly, in allowing the content of anxiety‐provoking experiences to vary across participants while categorizing these experiences as the “most worried about” anticipated experiences for each participant, this approach may increase the generalizability and ecological validity of findings related to these features and associations with anxiety.

First, we tested the hypothesis that anxious youth will show greater negative expectancy biases and higher magnitude of worry preceding real‐world anxiety‐provoking experiences, compared to non‐anxious youth. In line with past work on sex and gender differences, we hypothesized that anxious girls would report higher rates of worry about future experiences and predict more negative outcomes related to those experiences. We then examined whether attempted avoidance of these person‐specific experiences varied by diagnostic group (anxious, non‐anxious) and gender (girls, boys), hypothesizing that anxious girls would report the highest levels of attempted avoidance. Finally, we explored whether anticipatory ratings of negative expectancy or worry predicted attempted avoidance and whether these varied by diagnostic group and/or gender.

## METHOD

### Participants (Table 1)

**TABLE 1:** Demographics and clinical diagnoses

Variable	Anxious group			Healthy group		
	Female (*N* = 42)	Male (*N* = 28)	Total (*N* = 70)	Female (*N* = 31)	Male (*N* = 23)	Total (*N* = 54)
*Demographics*						
*Age (in years)*						
Mean	13.05	12.16	12.69	13.63	13.06	13.39
SD	2.85	2.53	2.74	2.52	3.04	2.74
% of prompts completed						
	73.36	71.09		82.64	76.19	
*Race*						
White						
N	31 (72.1)	18 (62.1)	49 (70.0)	17 (56.7)	14 (63.6)	31 (57.4)
Black/African American						
N	2 (4.7)	1 (3.4)	3 (4.3)	4 (13.3)	5 (22.7)	9 (16.7)
Asian						
N	1 (2.3)	3 (10.3)	4 (5.7)	3 (10.0)	0 (0)	3 (5.6)
American Indian or Alaskan Native						
N	0 (0)	0 (0)	0 (0)	0 (0)	0 (0)	0 (0)
Multiple races						
N	6 (14.0)	5 (17.2)	11 (15.7)	4 (13.3)	1 (4.5)	5 (9.3)
Unknown						
N	3 (7.0)	0 (0)	3 (4.3)	2 (6.7)	4 (17.4)	6 (11.1)
*Ethnicity*						
Latino or Hispanic						
N (%)	8 (18.6)	3 (10.3)	11 (15.7)	1 (3.3)	1 (4.5)	2 (3.7)
Not Latino or Hispanic						
N (%)	33 (76.7)	23 (79.3)	56 (80.0)	27 (90.0)	21 (95.5)	48 (88.9)
Unknown						
N (%)	0 (0)	3 (10.3)	0 (0)	2 (6.7)	2 (6.7)	4 (7.4)
*Clinical diagnosis*						
Generalized anxiety disorder						
N (%)	41 (97.6)	23 (82.1)	64 (91.4)			
Social anxiety disorder						
N (%)	32 (76.2)	19 (67.9)	51 (72.9)			
Separation anxiety disorder						
N (%)	14 (33.3)	10 (35.7)	24 (34.3)			
Specific phobia						
N (%)	12 (28.6)	9 (32.1)	21 (30.0)			
Panic disorder w/Agoraphobia						
N (%)	1 (2.4)	2 (7.1)	3 (4.3)			
Selective mutism						
N (%)	2 (4.8)	1 (3.6)	3 (4.3)			
Attention deficit Hyperactivity disorder						
N (%)	1 (2.4)	3 (10.7)	4 (5.7)			

Youth ages 8–18 years were recruited from the Washington DC area to participate in ongoing studies in the laboratory via flyers distributed to local schools and pediatricians' offices. Participants were invited to enroll in the protocol if they were seeking treatment for one or more anxiety disorders or were free of DSM‐5 diagnoses. Interested participants and their parents completed a screening visit during which a trained clinician administered a semi‐structured clinical interview to assess current and past diagnoses (K‐SADS; Kaufman et al., 1997). The K‐SADS contain child and parent interviews, and both were administered in our protocol, as is standard for the assessment. Interviews were performed separately by the same clinician. Thus, diagnoses reflect parent‐ and child‐reported symptoms. Participants were not eligible to participate if they met criteria for a mood disorder. From the larger laboratory protocol, 134 youth opted to participate in the EMA study. Of the 134 participants, 10 failed to complete the minimum number of prompts required for inclusion (<7 (33%) of prompts) and were excluded from analyses. Of the remaining 124 remaining participants, 70 met DSM‐5 criteria for at least one anxiety disorder (“anxious group,” 42 female participants, age in years *M* = 12.69, *SD* = 2.74). Besides anxiety, the only additional diagnosis present in the anxiety group was ADHD (*N* = 4). All participants were unmedicated. The remaining 54 youth were free of any psychiatric illness (“healthy group,” 31 female participants, age in years *M* = 13.39, *SD* = 2.74). Complete demographic information is presented in Table 1.

The anxious and healthy groups did not differ in age (*t*(122) = −1.18, *p* = .24, *d* = 0.24) or gender (*X* ^2^(1) = 0.05, *p* = .82). In line with the diagnostic categories, the anxious group endorsed more anxiety symptoms than the healthy group (self‐reported: *t*(117) = 10.20, *p* < .001, *d* = 2.07; parent‐reported: *t*(118) = 13.18, *p* < .001, *d* = 2.63).

We also tested gender differences within groups. There were no gender differences in the healthy group for age (*t*(50) = 0.33, *p* = .74, *d* = 0.09), or anxiety severity (self‐reported: *t*(47) = 0.73, *p* = .47, *d* = 0.22; parent‐reported: *t*(47) = 1.70, *p* = .10, *d* = 0.51). Within the anxiety group, age (*t*(70) = 1.24, *p* = .22, *d* = 0.30), did not vary by gender. However, in line with past research, male participants endorsed fewer anxiety symptoms (i.e., lower scores on the Screen for Child Anxiety Related Emotional Disorders, SCARED; Birmaher et al., 1997) than female participants (*t*(70) = 4.00, *p* < .001, *d* = 0.96). Notably, there were no gender differences in parent ratings of the youths' anxiety levels (*t*(70) = 0.22, *p* = .83, *d* = 0.05). There were also no gender differences in anxiety diagnoses (*p*s > .05, see Table 1) or total number of diagnoses met (*t*(70) = −0.38, *p* = .71, *d* = 0.19).

### Procedure

All procedures were approved by the National Institute of Mental Health Institutional Review Board. Prior to participation, parents and participants provided written consent/assent. During the screening process, parents were asked to report on their child's gender identity, with options for “male,” “female,” “neither exclusively male or female,” “other,” or “transgendered.” Parents of participants in the current analyses identified all participants as either male or female. During each laboratory visit, an assessment battery was completed by participants and their parents, which included the SCARED (Birmaher et al., 1997) to assess anxiety symptoms. For the current study, the SCARED scores collected closest in time to the EMA session were used. This could occur before or after the EMA session. All were collected within 3 months of study participation, and all occurred prior to the start of treatment. Anxious participants also received treatment following participation (i.e., manualized CBT [Walkup et al., 2008] and/or SSRIs).

Participants completed seven consecutive days of EMA on a lab‐provided or personal smartphone. Three times per day (morning, afternoon, evening) participants were prompted on their smartphone to answer a battery of questions (described below). Prompt times were randomized within 1‐hour blocks that participants prespecified via a self‐ and/or parent‐completed form based on their waking times, school schedules, and bedtimes. Participants were given 1 hour to complete each prompt; otherwise, that survey expired and data for that prompt were coded as missing. Participants were incentivized to complete as many prompts as possible by receiving a $10 bonus for completing over 75% of prompts (*M* = 75.69%, *SD* = 16.81%) and up to $100 total for participating ($75 for participation, $10 for completing 75% of the prompts, $15 for returning lab‐provided smartphones^2^). There was a difference in prompt completion rate between anxious and healthy youth (*t*(122) = −2.50, *p* = .01, *d* = 0.46), with healthy participants completing more prompts (*M* = 79.89%, *SD* = 17.82%) than anxious participants (*M* = 72.45%, *SD* = 14.55%). Importantly, the percentage of prompts completed did not differ by gender across groups (*t*(122) = 1.28, *p* = .20) or within groups (anxious: *t*(68) = 0.52, *p* = .61; healthy: *t*(52) = 1.64, *p* = .11). Further, the percentage of prompts was not related to self‐reported anxiety severity (SCARED total child‐report; *r* = −0.05, *p* = .59) or age (*r* = 0.078, *p* = .39).

The day before starting the EMA protocol, participants completed a 45‐minute training where research assistants walked through all EMA procedures (i.e., how to log in to the assessments, how to submit the assessment, etc.) and questions with participants, explaining all vocabulary and providing examples for relevant questions to reduce variability in participants' understanding of the EMA items. At this time, participants and their parents completed a form specifying 1‐hour windows when EMA prompts could be completed to avoid activities and coordinate with sleep and wake times for the following week.

EMA items featured in the current analyses assessed the event or concern that the participant was ***most*** worried about happening that day. In the morning prompt, participants were asked to think about and describe the thing they were most worried about happening that day (“*What are you* ***most*** *worried about happening today?*”). Worry was defined as “The feeling when you have something coming up and you may spend some (or lots) of time thinking about bad things that may happen.” Participants wrote out (in a free text response box) what that worry was and were then asked several follow‐up questions, including the type of worry (e.g., “*My family*,” “*My friends or peers*,” “*School*,” etc.; See Supplemental Materials for frequency of responses), magnitude of worry (“*How worried are you about this?*”), and anticipated severity of the experience (“*How bad do you expect it to be?*”). Here, anticipated severity of the experience serves as our measure of negative expectancy. Anticipatory ratings of worry and severity were rated on a five‐point Likert scale (1 = *Not at all*, 5 = *Extremely*). Free response answers where participants wrote “Nothing” or “None” were excluded from the analysis (range = 0–7, *M* = 1.04, *SD* = 1.79). Five participants answered “Nothing” or “None” at every prompt and were therefore excluded from analyses.

During the afternoon and evening assessments, participants were asked whether or not the thing they were worried about that morning happened (“*Did the thing you were worried about earlier happen?*”, binary choice: “*Yes*” or “*No*”). If the experience did happen, they were asked how bad the experience was (“*How bad was it?*”; five‐point Likert scale: 1 = *Not at all*, 5 = *Extremely*). Participants were also asked whether they attempted to avoid it happening (“*Did you do anything to try and avoid the situation?*”, binary choice: “*Yes*” or “*No*”). Avoidance in this assessment was defined as overt, or behavioral, avoidance. During training, all participants were given examples of avoidant behaviors (e.g., “You stayed home from school because you were worried or scared about getting a bad grade. Or if you didn't go on a tough bike trail because you were worried you might fall off your bike.”) and asked to share their own example with the research assistant to ensure they understood the concept. All assessments were administered via ReTAINE software (www.retaine.org).

### Data analysis plan

Due to the nested nature of the data (prompts within participants), multilevel modeling (MLM) was performed in R v3.6.0 using lme4 for linear models and the glmer option within lme4 for logistic models (Bates et al., 2014). Diagnostic group and gender variables were both dummy‐coded such that the reference group was healthy, male participants. All continuous Level‐1 predictors were person‐centered and continuous Level‐2 predictors were grand‐mean centered. In all models, the slopes were fixed with random intercepts. Random slopes were tested but did not significantly improve the models; thus, fixed slopes were used in all models. All analyses controlled for participant age.

### Associations among morning (i.e., anticipatory) EMA ratings, diagnostic group, and gender

Here, we explored potential interactions among anticipatory EMA ratings, diagnostic group (anxiety, healthy), and gender (male, female). Each rating (anticipated worry, anticipated severity) was run in a separate model (lme). In each model, diagnostic group, gender, and their interaction were fixed, dichotomous predictors and EMA‐reported rating was the dependent variable.

### Relations among afternoon and evening EMA ratings, diagnostic group, and gender

To test whether reports of the most‐worried‐about event occurring and attempted avoidance vary by diagnostic group and gender, logistic MLMs (glmer) were run. In each model, diagnostic group, gender, and their interaction were fixed, dichotomous predictors and EMA‐reported dichotomous response was the dependent variable. Models testing group by gender interactions for severity ratings were identical to the models run on the anticipatory ratings, such that group and gender were fixed, dichotomous predictors and EMA‐reported rating was the dependent variable.

### Predictors of attempted avoidance: Relations among anticipatory (morning) ratings and attempted avoidance

Next, logistic MLMs were used to test temporal associations among morning and afternoon ratings. We tested whether morning ratings of worry and anticipatory severity were associated with afternoon/evening endorsements of attempting to avoid the most‐worried‐about experience. Lagged (t−1) ratings of anticipatory worry and anticipatory severity were continuous predictors (in separate models). Dichotomous (yes or no) responses of attempted avoidance was the dependent variable. Lagged ratings were constrained to the same day and did not extend into the following day. If both afternoon and evening prompts were completed, anticipatory (i.e., morning) ratings were lagged to both (*N* = 55), allowing us to capture worries that could occur at either or both prompts. For instance, some reported worries were specific to one event (e.g., taking a test, trying out for a team), while others represented sustained worries/experiences throughout the day (e.g., throwing up, interacting with peers). Finally, we added both diagnostic group and gender as fixed predictor variables to the model to test whether relations among morning ratings and attempted avoidance varied by group, gender, or their interaction.

## RESULTS

### Associations among morning (i.e., anticipatory) EMA ratings, diagnostic group, and gender (Figure 1)

**FIGURE 1:**
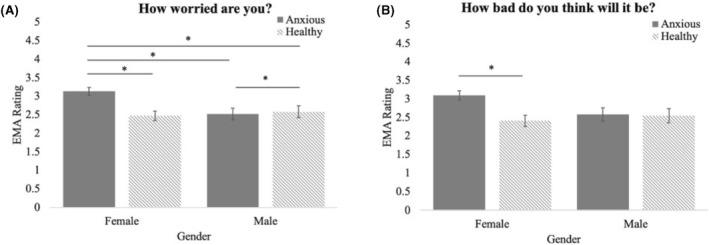
Associations among morning (i.e., anticipatory) EMA ratings, diagnostic group, and gender. MLMs showed significant diagnostic group by gender interactions for (A) magnitude of worry, and (B) negative expectancy in anticipation of anxiety‐provoking experiences in vivo. *p < .05. For illustrative purposes, graphs represent mean ratings for each participant type (i.e., diagnostic group by gender) collapsed across prompts.

Models examining anticipatory worry and severity yielded significant group by gender interactions (worry: *b* = 0.71, *SE* = 0.28, *t* = 2.55, *p* = .01; severity: *b* = 0.67, *SE* = 0.33, *t* = 2.04, *p* = .04). Follow‐up tests demonstrated that the interaction for magnitude of worry was driven by higher worry among anxious girls as compared to the other groups, *p*s < .05 (See Figure 1A). In the severity (i.e., negative expectancy) model, anxious girls reported significantly higher anticipated severity healthy girls (*t*(108) = 3.59, *p* = .002) but did not differ from either group of boys (anxious: *t*(108) = 2.40, *p* = .08; healthy: *t*(108) = 2.45, *p* = .07; See Figure 1B). Age was not significant in any models.

### Relations among afternoon and evening EMA ratings, diagnostic group, and gender (Figure 2)

**FIGURE 2:**
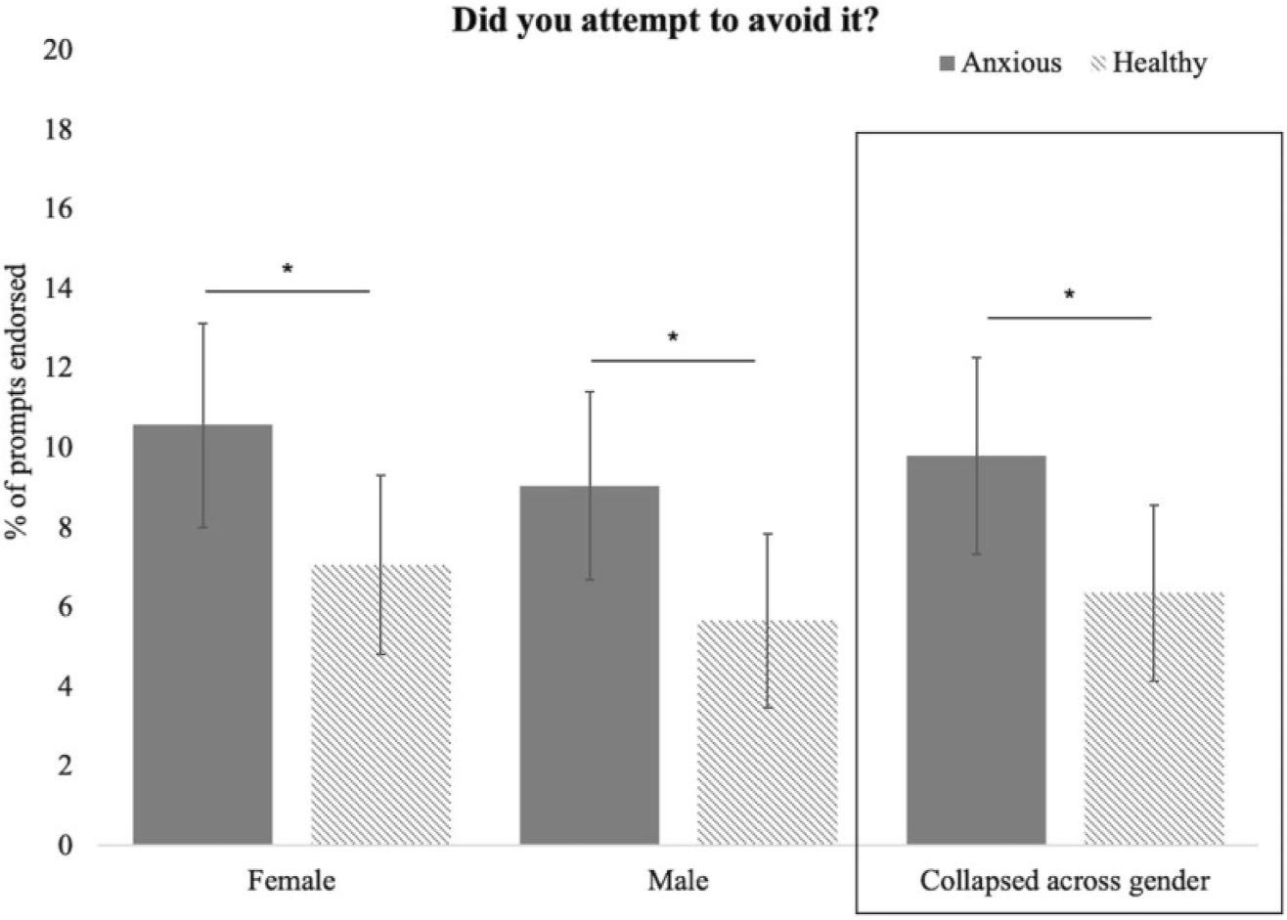
Relations among afternoon and evening EMA ratings, diagnostic group, and gender. The diagnostic group by gender interaction was not significant for attempted avoidance of anxiety‐provoking experiences. Instead, there was a significant main effect of diagnostic group. *p < .05. For illustrative purposes, graphs represent mean percentage of prompts endorsed for each participant type (i.e., diagnostic group by gender) collapsed across prompts.

A significant diagnostic group by gender interaction emerged from the model examining whether the experience occurred (*b* = 0.79, *SE* = 0.40, *z* = 1.95, *p* = .05). Follow‐up tests showed that healthy girls reported higher rates of the experience occurring. Similarly, when the event did occur, there was a significant group by gender interaction in severity ratings of the experience (*b* = 0.93, *SE* = 0.46, *t* = 2.00, *p* = .05). Patterns suggest that anxious girls reported the experience as more severe than other groups; however, no post‐hoc contrasts reached significance. Finally, there was no interaction between diagnostic group and gender for attempted avoidance of the experience (*b* = −1.22, *SE* = 1.24, *z* = −0.98, *p* = .32). Instead, there was a main effect of diagnostic group with anxious youth reporting higher rates of attempted avoidance regardless of gender (*b* = 1.86, *SE* = 0.49, *z* = 3.8, *p* = .0001; Figure 2). Age was not significant in any models.

### Predictors of attempted avoidance: Relations among anticipatory (morning) ratings and attempted avoidance (Table 2)

**TABLE 2:** Associations among morning ratings of “most worried about” event and PM ratings

Predictors	What are you most worried about happening today?	
	How worried are you about this?	How bad do you expect it to be?
*Did the thing you were worried about earlier happen?*		
*b (SE)*	0.08 (0.14)	−0.12 (0.12)
*Z*	0.63	−1.02
*P*	.47	.35
*If it did happen…How bad was it?*		
*b (SE)*	0.12 (0.09)	0.24 (0.07)
*T*	1.30	3.35***
*P*	.25	.002
*Did you try and do anything to avoid it?*		
*b (SE)*	0.49 (0.21)	−0.17 (0.18)
*Z*	2.37*	−0.98
*P*	.02	.33

*Note*: The left hand column indicates the variable that is being predicted. Prompts where participants responded “Nothing” to the question of “what are you most worried about happening today?” were removed from the current analyses. This resulted in a total of 5 participants being removed from the analyses.

*p < 0.05, **p < 0.01, ***p < 0.001.

Lagged analyses indicated that greater anticipatory worry, but not predicted severity, was associated with more attempted avoidance. However, additional analyses indicated that the strength of the relation between anticipatory worry and attempted avoidance did not differ by diagnostic group or gender. Age was not significant in any models of avoidance.

## DISCUSSION

Using a novel EMA assessment, the present study demonstrates that clinically anxious youth endorsed more negative expectations and higher magnitude of worry surrounding anticipated real‐world anxiety‐provoking experiences. This association was particularly evident in anxious girls who reported the highest levels of worry and expected future experiences to be the most negative. Further, compared to non‐anxious youth, clinically anxious youth reported higher levels of attempted avoidance. Importantly, unlike for negative expectations or worry, there were no gender differences in rates of attempted avoidance among anxious youth. Finally, magnitude of worry, but not anticipated severity, predicted attempted avoidance, in a way that also did not vary by gender. These findings extend current theories regarding the interplay of negative expectations and magnitude of worry for anticipated future experiences, and attempted avoidance to pediatric anxiety and naturalistic experiences. By using EMA to focus on person‐specific anxiety‐inducing experiences, we were able to begin to understand how these processes and experiences unfold in the real world.

Using EMA, we demonstrated that morning ratings of expected severity and magnitude of worry surrounding an anxiety‐provoking experience were significantly higher in anxious, compared to non‐anxious, youth. These findings replicate established findings in anxious adults and available data in anxious youth (e.g., De Jong & Muris, 2002; Guyer et al., 2008; Smith et al., 2018) and serve as validation of the present EMA protocol for use in pediatric populations. Similarly, we found significant gender differences in these anticipatory processes, with anxious girls reporting significantly more severe worry in anticipation of anxiety‐provoking experiences compared to the other groups (anxious boys, non‐anxious girls and boys; Behnke & Sawyer, 2000; McLean & Hope, 2010; Straube et al., 2009). Somewhat surprisingly, anxious girls reported more negative expectations, but only compared to non‐anxious girls, not to anxious nor non‐anxious boys. Interestingly, anxious males demonstrated a different pattern of reported symptoms than anxious females, reporting higher anticipatory worry, but not expected severity, compared to non‐anxious males, and not differing from non‐anxious females on either anticipatory rating. Varying patterns of anticipatory ratings in anxious boys and girls suggest a nuanced view of potential gender differences in anxiety‐related processes. Specifically, heightened anticipatory worry may be particularly important to understand and target therapeutically in anxious females.

With the current assessment, we are not able to determine whether greater magnitude of worry and more negative expectations in anxious girls are results of symptom‐reporting biases or underlie a true difference in the experience of anxiety. However, given that the anticipatory ratings occur in the morning, prior to the probed anxiety‐provoking experience occurring that day, there are no concerns regarding biases in retrospective reporting of those events. Current criteria for pediatric anxiety disorders emphasize the presence of psychological processes, such as distress, and behaviors, such as stomach aches or crying, that manifest when children and adolescents anticipate confronting a feared stimulus or experience (Pine & Klein, 2015). However, as demonstrated here, magnitude of anticipatory worry and self‐reported anxiety symptoms (SCARED‐Child) are more highly endorsed by anxious girls, despite there being no differences in anxiety diagnoses or parent‐reported symptoms in the anxious girls and boys (See Table 1). We have demonstrated this informant discrepancy in previous work using EMA in youth with anxiety (Smith et al., 2019), although not by gender. Heightened anticipatory worry in anxious girls, compared to anxious boys, may be explained by findings that girls experience anxiety as more distressing than boys (Altemus et al., 2014). It is important to note that posthoc tests for the negative expectancy model demonstrated that, compared to non‐anxious girls, anxious girls expected the experience to be more severe, but ratings did not differ from those of anxious boys. Together, these findings suggest that there may be gender‐specific and gender‐non‐specific associations between anxiety and anticipatory processes.

If we assume these represent true differences in how anxious boys and girls experience anxiety, rather than biases in reporting, there are several explanations for why these differences may exist. First, we cannot rule out the possibility that anxious males are underreporting anticipatory symptoms or that anxious females are overreporting their anxiety symptoms, especially since parent‐reported and clinician‐rated anxiety did not differentiate by gender. Another potential explanation is that anxious girls have different ways of handling feelings of anxiety and worry than anxious boys, which increase the subjective intensity of those worries as reflected in rating scales. For instance, compared to boys, girls often report engaging in more rumination, or deep and often negative repetitive thoughts, about distressing experiences (Johnson & Whisman, 2013; Nolen‐Hoeksema & Jackson, 2001). On the other hand, anxious boys report using cognitive distraction techniques to a greater extent than girls do (Stone et al., 2019). Past work has demonstrated that repetitive, negative thoughts prolong anxious mood (Blagden & Craske, 1996) and predict anxiety symptoms (Calmes & Roberts, 2007; McEvoy et al., 2019; Young & Dietrich, 2015). Among anxious girls, repetitive thinking about these experiences may result in more prolonged, and intense, feelings of anxiety that are then captured in the retrospective reports. This may be particularly evident in our finding of heightened anticipatory worry in anxious girls, compared to anxious boys. Repetitive negative thoughts surrounding an anticipated event may increase anticipatory‐related distress or worry in anxious females to a greater extent than it does anxious males. If anxious boys are engaging in more cognitive distraction techniques, then this may serve to decrease the subjective intensity of the worries themselves, as reflected in lower anticipatory anxiety scores. The current paradigm did not assess strategies for handling anxiety; however, this work would benefit from exploring these as potential mechanisms underlying the gender differences identified in this study.

When avoidance of these anxiety‐provoking experiences was probed, anxious youth reported higher rates of attempted avoidance compared to non‐anxious youth. There were no gender differences in attempted avoidance, despite anxious girls reporting more negative expectations and higher worry regarding these experiences. This finding is inconsistent with past work demonstrating greater avoidance in female participants (McLean & Hope, 2010; Stoyanova & Hope, 2012). However, the current study is the first to utilize individualized anxiety‐provoking experiences assessed in vivo. It is possible that while women/girls learn to avoid more quickly and exhibit avoidant behaviors at a higher rate than men/boys do in the laboratory (McLean & Hope, 2010; Stoyanova & Hope, 2012), avoidance of real‐world experiences that are particularly anxiety‐provoking to individual adolescents is comparable across genders. Or, anxious boys experience different types of anxiety‐provoking experiences in the real‐world, which are not adequately captured in laboratory tasks of avoidance behaviors or in retrospective reports. This difference may also reflect differences in avoidance strategies between anxious boys and girls.

It is also possible that gender differences may depend on the type of avoidance that is measured. In the current paradigm avoidance was operationalized as overt, or behavioral, avoidance. This means that participants made a physical or behavioral effort to not experience the specified event. Such overt avoidance is predictive of both anxiety and depression (Grant et al., 2013; Panayiotou et al., 2014, 2017), but does not capture internal avoidance strategies such as cognitive avoidance. While we measured overt avoidance here, it is possible, given purported gender differences in cognitive coping strategies among anxious girls and boys, that there are gender differences in some, but not all, types of avoidance. Further, we probed the experience youth were *most* worried about happening that day. It is possible that there are no differences in avoidance between girls and boys at extreme levels of worry experiences. Finally, given overall low rates of reported avoidance, we cannot rule out that the c analysis is underpowered to detect such effects. More work is needed to replicate these findings and explore potential underlying causes of discrepancies in gender differences between laboratory‐based and real‐world measures of avoidance.

While the current findings need to be replicated and hypothesized explanations need to be tested, the findings in the current paper have important research and treatment implications. If replicated, the fact that anxiety by gender interactions emerge in anticipatory processes, but not in attempted avoidance, provides an exciting avenue for future research exploring potential predictors of avoidance that may vary by gender. Here, we demonstrate that magnitude of worry, but not negative expectancy (i.e., anticipated severity), predicts whether youth will attempt to avoid a potentially negative experience. While magnitude of worry predicted attempted avoidance, this association did not vary by diagnostic group or gender. This may be due to overall low rates of avoidance, resulting in insufficient power to detect the full interaction. It is also possible that other factors, such as context or opportunity, are greater predictors of avoidance than motivating factors such as worry and expected severity. For instance, it is much easier to avoid going to watch a football game with peers than it is to skip a group presentation in history class, even if levels of worry are the same. It is also important to note that these ratings reflect feelings about the event youth were most worried about occurring that day (in the morning) and may not have been the most anxiety‐provoking event that occurred that day (in hindsight). Thus, if we probed the most anxiety‐provoking experience for severity and avoidance, different patterns may have emerged. Understanding precursors, or potential predictors, of avoidance behaviors and if they vary by gender, may help shed light on potential therapeutic targets aimed at reducing avoidance in youth with anxiety disorders. For instance, the current study suggests that magnitude of worry predicts attempts to avoid, therefore cognitive treatments aimed at reducing the magnitude of worry may be particularly effective in participants who endorse high anticipatory worry (e.g., Topper et al., 2017). Importantly, the EMA paradigm itself may be used as an intervention tool when participants endorse extreme worry regarding an anticipated event that day (e.g., Pramana et al., 2014).

There are several limitations present in the current manuscript that future work could address. First, there were differences in completion rates between the anxious and non‐anxious groups. The fact that non‐anxious youth completed more prompts than anxious youth was unexpected, and we cannot rule out that failure to complete prompts is itself a form of avoidant behavior. However, the finding that avoidance rates are higher in the anxious group despite the lower completion rates suggests that this effect would hold up even if the groups were matched on completion rates. A second limitation is that the current EMA paradigm still contains self‐reported information. While the information collected is closer in time to the experience and thus assumed to be more accurate, there may be experiences, particularly of avoidant behaviors, that participants are unable to remember or unwilling to report. Future work would benefit from validation with non‐self‐report measures, such as behavioral observations or passive sensing. Similarly, due to limitations of the program software, we were unable to remind participants in the PM prompts what they reported in the AM prompts. Given that participants noted these events as their most anticipated worrisome event, we are confident that they were able to recall the event specified in the morning but are unable to confirm this in the current dataset. Further, the relatively small sample size and large age range may limit the implications and generalizability of the current findings. Future studies in a limited age range or larger sample in the current age range would provide adequate power to test the role of normative development in these associations. Finally, gender in the current study was parent‐reported. While the question regarding gender did include non‐binary response options (e.g. “neither exclusively male or female,” “other,” or “transgendered”) we cannot ascertain that parent‐reported gender was an accurate reflection of the child's gender identity.

Despite these limitations, these findings underscore the importance of understanding how anxiety‐relevant processes unfold in the real world and how these experiences may differ in adolescent girls and boys. It also highlights the importance of assessing person‐specific experiences using temporally sensitive methods, such as EMA. Every adolescent has different anxiety‐provoking experiences and capturing shared and unique features of those experiences across anxious youth is critical for identifying generalizable therapeutic targets.

## AUTHOR CONTRIBUTION

All authors contributed to the conception and design of the study. Emily Jones, Anni Subar, and Quyen Do collected the data included in the manuscript. Ashley Smith analyzed the data. Ashley Smith, Emily Jones, Daniel Pine, and Jennifer Silk contributed to the interpretation of the data. Ashley Smith, Emily Jones, Daniel Pine, and Jennifer Silk drafted the manuscript. All authors provided significant input on all aspects of the study design and manuscript preparation. All authors have read and approved the final version of the manuscript.

## CONFLICT OF INTEREST

The authors have declared that they have no competing or potential conflicts of interest.

## ETHICS STATEMENT

Informed consent and assent were obtained from all parental guardians and children, respectively, and the study was approved by the National Institutes of Health Institutional Review Board (study number 01M0192).

## Supporting information

Supplymentary Information 1

## Data Availability

The data that support the findings of this study are available on request from the corresponding author. The data are not publicly available due to privacy or ethical restrictions.

## ENDNOTES

^1^
The authors acknowledge that historically “sex” and “gender” were not consistently defined or used in the literature examining differences between anxious females and males. For consistency we utilize “gender” rather than “sex” throughout the manuscript while acknowledging that the original articles cited may vary in their use of “sex” and “gender” and in most cases did not clearly assess gender identity as opposed to sex assigned at birth.

^2^
All participants received the $15 compensation for either utilizing their own smartphones or returning lab‐provided phones.
