# Supplementary material for: The role of anxiety and gender in anticipation and avoidance of naturalistic anxiety‐provoking experiences during adolescence: An ecological momentary assessment study
Source: JCPP Adv. 2022 Jun 22;2(3):e12084. doi: 10.1002/jcv2.12084 (PMC10242842; doi:10.1002/jcv2.12084)
Supplement: Supplementary file 1 — Supplymentary Information 1 [file JCV2-2-e12084-s001.docx]

**Supplemental Materials**

**Participants**

**Race and Ethnicity**

The anxious and healthy groups did not differ in race (*X*^2^(5)=7.38, *p*=.19). However, the groups did differ in ethnicity, *X*^2^(1)=4.47, *p*=.03, where the anxious group had significantly more Latino/a and Hispanic participants compared to the healthy group.

There were no gender differences in the healthy group for race (*X*^2^(4)=4.07, *p*=.40) or ethnicity (*X*^2^(1)=0.03, *p*=.86). Within the anxiety group, race (*X*^2^(5)=5.56, *p*=.35), and ethnicity (*X*^2^(1)=0.74, *p*=.39)) did not vary by gender.

**Results**

**Type of Worry**

Participants are asked to include a description of the “most worried about” experience in a free response box and to categorize it into 1 of 8 potential categories (“my family,” “my friends or peers,” “people I don’t know well,” “school,” “sports or extracurricular activities,” “my health and safety,” “Nothing in particular,” and “Other.”)

**Certainty that the “most worried about” experience will occur**

As part of the morning ratings, participants were also asked “*how certain are you that it will happen*” and responded on a 5-point Likert scale from Not at all to Extremely. We ran the same model as the other anticipatory ratings (i.e., Group and Gender predicting certainty, controlling for Age) with this question and found no effects of Group (*b* =-0.03, SE=.22, *t*=-0.11, *p*=.91), Gender (*b* =0.27, SE=.26, *t*=1.04, *p*=.30), or Group X Gender interaction (*b* =-0.33, SE=.38, *t*=-0.88, *p*=.38).

Using the same lagged models described in the manscript methods section, we tested whether level of certainty in the morning, predicted whether the event occurred and attempted avoidance of the event. The models indicate that rating of perceived certainty that the event will occur (in the morning) does predict whether it occurs (*b* =0.32, SE=.10, *t*=3.34, *p*=.0009) but does not predict attempted avoidance (*b* =0.16, SE=.15, *t*=1.06, *p*=.29) of that experience.
